# CHANGES IN PERSONALITY TRAITS PREDICT ALLOSTATIC LOAD

**DOI:** 10.1093/geroni/igae098.2501

**Published:** 2024-12-31

**Authors:** Jacob Alderson, Sarah Miller, Nicholas Turiano

**Affiliations:** West Virginia University, Morgantown, West Virginia, United States; West Virginia University, Morgantown, West Virginia, United States; West Virginia University, Morgantown, West Virginia, United States

## Abstract

Understanding the influences personality change has on physical health has been identified as a key direction for personality research (Leszko et al., 2016). One key indicator of health and aging process is allostatic load, an objective index capturing physiological regulation (or dysregulation) across a variety of biological systems (e.g., inflammation, cardiovascular health). While some evidence suggests cross sectional associations between personality traits and allostatic load in older adult populations (Yoneda et al., 2023), little research at present has assessed if changes in personality are also associated with allostatic load. The present study utilizes the Midlife Development in the United States (MIDUS) data set (N = 998; Mean age = 46.14; range = 25-74), to analyze change scores in personality traits at two time points (1994-1995 to 2004-2005) as predictors of allostatic load at a subsequent time point (2004-2009). Age, race, gender, education level (at 1994-1995) and baseline level of personality traits were used as covariates. Regression analyses revealed that increases in openness (b = -.20, p =.034) and decreases in agreeableness (b =.27, p =.004) were associated with a lower allostatic load. These findings have implications for considering the role personality development has on healthy aging. Given that naturalistic change in personality does associate with important markers of physical health and physiological processes, future studies could attempt to intervene at the level of personality (e.g., attempt to increase openness) in order to promote more optimal physical health and aging processes.

